# Critical Review of Selected Analytical Platforms for GC-MS Metabolomics Profiling—Case Study: HS-SPME/GC-MS Analysis of Blackberry’s Aroma

**DOI:** 10.3390/foods13081222

**Published:** 2024-04-17

**Authors:** Jovana Ljujić, Ljubodrag Vujisić, Vele Tešević, Ivana Sofrenić, Stefan Ivanović, Katarina Simić, Boban Anđelković

**Affiliations:** 1 Faculty of Chemistry, University of Belgrade, Studentski trg 12–16, 11000 Belgrade, Serbia; 2 Institute of Chemistry, Technology and Metallurgy, National Institute of the Republic of Serbia, University of Belgrade, Njegoševa 12, 11000 Belgrade, Serbia

**Keywords:** multivariate data analysis, metabolomics platforms, headspace solid-phase micro extraction, volatile compounds, gas chromatography mass spectrometry, blackberries aroma

## Abstract

Data processing and data extraction are the first, and most often crucial, steps in metabolomics and multivariate data analysis in general. There are several software solutions for these purposes in GC-MS metabolomics. It becomes unclear which platform offers what kind of data and how that information influences the analysis’s conclusions. In this study, selected analytical platforms for GC-MS metabolomics profiling, SpectConnect and XCMS as well as MestReNova software, were used to process the results of the HS-SPME/GC-MS aroma analyses of several blackberry varieties. In addition, a detailed analysis of the identification of the individual components of the blackberry aroma club varieties was performed. In total, 72 components were detected in the XCMS platform, 119 in SpectConnect, and 87 and 167 in MestReNova, with automatic integral and manual correction, respectively, as well as 219 aroma components after manual analysis of GC-MS chromatograms. The obtained datasets were fed, for multivariate data analysis, to SIMCA software, and underwent the creation of PCA, OPLS, and OPLS-DA models. The results of the validation tests and VIP-pred. scores were analyzed in detail.

## 1. Introduction

Metabolomics has been employed in a variety of applications, including the discovery of biomarkers and enzymes in food and nutrition, plant biotechnology, and health. Metabolomics is described as the comprehensive, simultaneous examination of many metabolites in biological systems, and it has emerged in a wide range of research areas. Huge progress in this area has been aided by the development of high-resolution analytical techniques such as nuclear magnetic resonance (NMR) and mass spectrometry (MS), which allow for the examination of a wide range of metabolites at various concentration levels. Multivariate data analysis is an essential tool for the analysis of large and complex data sets [1,2]. With this approach, the analysis of large and complex data sets is feasible [3]. It was developed in parallel with the development of computation and computers. In order to adequately process a large data set, such as the metabolome, the application of multivariate analysis is necessary. Additionally, the large number of MS databases that make it easier to analyze and identify compounds greatly facilitates this process. The metabolome is very complex, and therefore there is a justified need to develop methods that will facilitate its interpretation and processing in order to discover metabolites of importance [4,5,6]. One of the most important steps is the initial processing of raw data in order to obtain a reliable model using a multivariate analysis [7,8]. In addition, depending on the analytical technique used, it is necessary to establish procedures and workflows for research [9]. There are a number of platforms in use today that lead to the fast and efficient processing of large amounts of data, as well as their preparation for further metabolome analysis [10]. High resolving power, robustness, good reproducibility, selectivity, and high sensitivity are characteristics that make GC-MS an excellent analytical platform. Electron ionization (EI) is most often used because there are a large number of mass spectra libraries that facilitate the analysis. The results of GC-MS analysis consist of *m*/*z* values, retention times, and intensities of different peaks [11]. The fluctuations in the chromatograms’ retention times, which are particularly noticeable when a high number of samples is used, could be an issue [12]. Therefore, it is necessary to properly process the obtained raw data in order to obtain valid datasets [13]. Some of the basic steps are baseline corrections to define the area for peaks of importance, as well as alignment to avoid shifting retention times and to ensure the uniformity of the entire data set. There are a number of ways to process raw GC-MS results, using different platforms and software to prepare them for multivariate analysis [14,15].

In order to characterize cultivars, it is necessary to take into consideration the entire profile during the analysis in order to draw appropriate conclusions and understand the correlation of all metabolites in the metabolome [16]. The whole chromatogram (for all detected *m*/*z* values) is significant for nontargeted metabolomic investigations, which motivates attempts to choose experimental conditions that enhance metabolite peak accessibility [17]. Accurate analysis of all of these data is usually accompanied by some difficulties. It could be challenging to discern certain “real” chromatographic peaks from noise. Sometimes the separate MS scans contain peaks from coeluting mixtures of metabolites that are not chromatographically separated. Hence, peak enumeration which separates “true” peaks from noise in a chromatogram, and spectral deconvolution which, according to the recent literature, is becoming more and more common, are the first processing steps that follow the storage of raw GC-MS data [18]. These steps yield putative pure spectra from two overlapping peaks. These procedures can be carried out either using commercial software designed for a particular manufacturer’s equipment or using freely accessible software such as AMDIS [19]. In previous works, the main focus of the researchers was on the dominant components of aroma. They observed them as markers for the recognition of certain types of fruit [20] or geographical origin [21]. In plant metabolomics of fruit varieties, differences in genetic variability can be ruled out due to the dominant vegetative method of propagation (cloning) [22]. However, differences in replicants of the same type of sample can often have bigger effects on the main compounds than differences between samples. This is mostly because of different levels of maturity or stress-causing environmental factors like sunlight, humidity, pathogen exposure, and so on [23]. For this reason, the scaling and preprocessing of data are equally as important as the degree of sensitivity and selectivity of the applied instrumental technique and the applied statistical platform [24,25].

Blackberries are widely consumed fruits that are employed in many different processed goods. Both consumers and food suppliers place a great deal of importance on fruit quality, with premium fruits typically having greater market potential. As a result, berry growers worldwide seek berries that are large, firm, flavorful, and nutrient-rich [26]. With their bioactive components, which include phenolic acids and flavonoids [27], they have strong antioxidant activity [28] and help prevent a variety of ailments. Up to now, blackberries have been the subject of extensive studies using liquid chromatography with mass spectrometry and multivariate data analysis [27,28,29,30]. To the best of our knowledge, this is the first time that a metabolomic approach has been used in the analysis of blackberry aroma, as well as the first time that the results of different platforms for preprocessing GC-MS results have been compared. 

This paper presents the influence of different online analytical platforms for data “extraction” and MestReNova software integration solutions on the results of a multivariate analysis for determining the aroma profile of different blackberry cultivars that were grown in the Zeleni Hit Company experimental field, near Belgrade. The headspace solid-phase microextraction coupled to the gas chromatography-mass spectrometry (HS-SPME/GC-MS) method was used to track the changes. Non-target quantitative component profiling, to investigate the aroma profiles of different blackberry cultivars, was used. Six blackberry cultivars, Columbia Star [31], Loch Ness [32], Nachez [33], Ouachita [34], Prime-Ark 45 [35], and Von [36], were analyzed on three different platforms: MestReNova 12.0 with automatic and manual corrections of detected peaks, XCMS online [37], and SpectConnect [38], in order to compare their results and identify an optimal solution for GC-MS data preprocessing. The criteria for comparison were the number of identified peaks, the quality of the peaks, and the results of statistical analyses. The peak numbers and identification were validated through the manual check of one representative chromatogram of each cultivar. In total, 269 compounds were detected and 216 were identified. The methylundecanoate was used as an internal standard for the normalization and quantification of each compound.

## 2. Materials and Methods

### 2.1. Sample Collection and Preparation

In July 2021, in the experimental field of the Zeleni Hit DOO (Batajnički put ZH, Belgrade 11080) company fruits were collected from the six blackberry varieties Loch Ness, Ouachita, Nachez, Von, Prime-Ark 45, and Columbia Star. The samples were stored in plastic sterile bottles at a temperature of −18 °C until analysis.

In headspace vials was placed 2 g from each sample, 100 mg of NaCl (Sigma-Aldrich, Saint Louis, MO, USA), and 1 μL of methyl undecanoate solution in dichloromethane (PolyScience Corp. Niles, IL, USA) with a concentration of 2 ppm. The vials were tightly closed and incubated in a water bath at 60 °C for 30 min. During incubation in the empty space of the vial, fiber emerged for the solid-phase microextraction with polydimethylsiloxane (PDMS) as an adsorbent. A manual SPME arrow injection kit was used for incubation and the injection of concentrated blackberries into the GC-MS inlet. After injection, the fiber was kept in the heated inlet for 20 s before starting the analysis for desorption, and for another four minutes after starting the analysis, to condition it for the next sample. Blank samples were measured every day before measuring berry samples.

### 2.2. GC-MS Analysis

The Agilent 7890B GC system (Agilent Technologies, Santa Clara, CA, USA) equipped with a 5977 mass selective detector (MSD) was used for aroma compound GC-MS analysis. For separation, a non-polar HP-5MSI capillary column (30 m × 0.25 mm, 0.25 μm film thickness) was used. The oven temperature was programmed to increase linearly from 60 °C to 240 °C at a rate of 3 °C/min. Helium was used as a carrier gas, inlet pressure was constant at 16.7 psi (flow 1.0 mL/min at 210 °C), and splitless mode was used. The MS range was 40–550 amu, the electron ionization energy was 70 eV at 230 °C, and the quadrupole temperature was 150 °C. The transfer line temperature was kept at 315 °C.

### 2.3. Data Processing

Library search and mass spectral deconvolution and extraction of the derivatized compounds were performed using the MSD ChemStation software, version E02.02 (Agilent Technologies, Santa Clara, CA, USA), the NIST AMDIS (Automated Mass Spectral Deconvolution and Identification System) software version 2.70, and the commercially available Adams04, NIST17, and Wiley07 libraries containing approximately 500,000 spectra. 

For the eXtensible Computational Mass Spectrometry (XCMS) online platform (Version 3.7.1), using the MSD ChemStation software, all the MS chromatograms were converted to the AIA format. Based on the R software, the peak picking, nonlinear peak alignment, and matching of the retention times were then carried out utilizing this platform [39,40]. Using the CentWave feature detection algorithm, the maximum allowed *m*/*z* deviation in consecutive scans was set at 100 ppm. The minimum and maximum chromatographic peak widths were set at 5 and 10 s, respectively. The minimum difference in *m*/*z* for peaks with overlapping retention times was set at 0.01 and the signal/noise threshold was set at 6. In order to create peak density chromatograms and group peaks across samples, 0.5 is the minimum fraction of samples required in at least one sample group in order for it to be a valid group. Ten seconds is the allowable retention time deviation for peak alignment. After being standardized to the content of the internal standard (methylundecanoate), the data in the table from the XCMS online platform were put through multivariate data analysis.

The SpectConnect (Version 1.0) online platform was used according to the instructions given in the paper by Styczynski et al. and in online instructions [15,38]. The AMDIS software (Version 2.73) was used for spectral deconvolution and data set extraction. 

The MesReNova 12.0 software was used for the automatic detection and integration of chromatographic peaks. Peaks were detected in the range from 2.9 to 45 min with the highest sensitivity (200), automatic smoothing, and no area threshold (0%). The obtained tables were merged into one dataset. The siloxane signals that are also present in the blanks were manually removed. DRS was used for the automatic identification of individual components in representative chromatograms of each cultivar. 

Finally, using the MesReNova 12.0 software, each chromatogram was manually checked, and the peaks that it did not recognize were additionally integrated. Every pick from one representative chromatogram from each cultivar was manually checked for MS fragmentation pattern matching as well as for retention indexes with NIST17 and Wiley07 library data. 

Multivariate data analysis was performed using SIMCA software (version 15, Umet-rics, Umeå, Sweden). The GC-MS data were mean-centered and scaled using the square root of the standard deviation as the scaling factor (Pareto). For the MesReNova data sets, Excel 16 was used for the normalization of the content of the internal standard (methyl-undecanoate).

## 3. Results and Discussion

Ten replicates (one berry in the stage of technological maturity) of each cultivar were analyzed using the headspace SPME GC-MS instrumental technique. The obtained data were processed using online platforms (SpectConnect and XCMS), with the fact that the SpectConnect platform provides the possibility of using different data sets to obtain models (relative abundance—RA, integrated signal—IS, and base peak—AM) and compare them with semimanual and manual processed data from the utilization of the MestReNova software. The PCA, PLS-DA, and OPLS-DA models were generated and the main statistical parameters of each of them are presented in Table 1. According to the initial PCA model cultivars, Loch Ness had the most unique data set; it has been separated most in relation to the others in the score plot. For this reason, it was chosen for comparison with the others by making individual pairs in OPLS-DA models. Each OPLS-DA model was validated with CV-ANOVA (see Table 1) and permutation test. In order to compare the results of those models, VIP-predictive plots were analyzed.

In comparison to SpectConnect (119) and XCMS (72), the highest number of volatile components (peaks) was observed, after manual evaluation of the chromatograms (167), as twice as much (87) as in automatic peak detection with the highest sensitivity. In the further detailed investigation of the Total Ion Chromatogram (TIC) using the AMDIS deconvolution algorithm, 269 compounds were detected. The 219 volatile components were identified, but, for 50 minor (less than 5%) compounds, it was not possible to accomplish an identification due to a low ion abundance (concentration) and/or the lack of reference spectra in the libraries and retention indexes (see Supporting Information Table S1).

By comparing the models obtained using different datasets and their major statistical parameters, it can be noticed that the highest coefficient of determination (R^2^) has a model with the XCMS data set and SpectConnect—base peak data set models with an R^2^ value of about 0.9. In the remaining applied models, the R² value was from 0.516 to 0.766. A similar trend was observed with the predictive ability of the models (Q^2^) (see Table 1). The major reason for that could be the number of variables as well as the data type that was used to generate the data set. Specifically, in the XCMS platform the abundance of the base ion was extracted instead of the total ion current. In that instance, the noise detection threshold is rather high, particularly for GC-overlapped analytes. This reduces the number of detected metabolites that would be subject to multivariate analysis but provides models with high statistical significance. Although it is quite intuitive and easy to use, this platform is not the most suitable for the metabolomic analysis of GC-MS results, where the electron impact ionization technique is used and plant extracts are the subject of analysis. On the contrary, the application of this platform to the analysis of compounds of similar polarity (e.g., aromas) gives reliable results [41]. 

**Figure 1:**
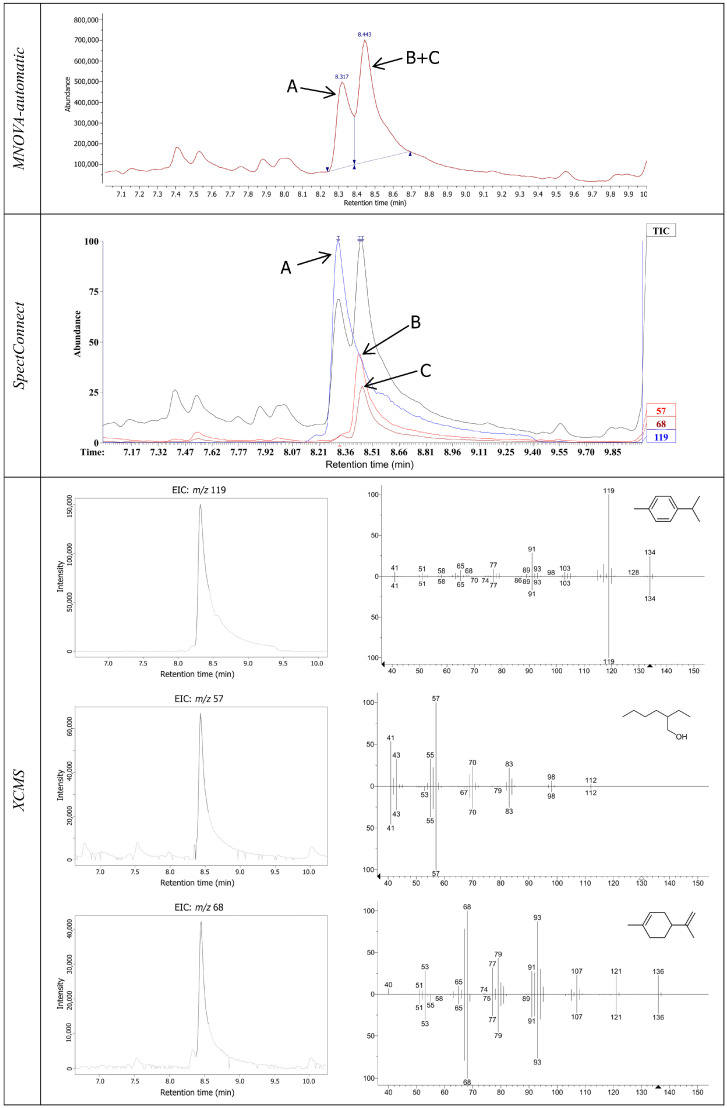
Expanded region of the SPME GC-MS chromatogram of the Ouachita cultivar sample with the results of the MestReNova, SpectConect (with AMDIS preprocessing), and XCMS software. A: *p*-Cymene, B: 2-Ethyl-1-hexanol, and C: Limonene.

To overcome the problems of peak overlapping, the SpectConnect platform used NIST software, which extracts individual component spectra from gas chromatography/mass spectrometry (GC-MS) data files by deconvolution and ion-counting noise procedures (Figure 1). As a result of the SpectConnect platform, three similar datasets based on relative abundance (RA), integrated signals (ISs), and base peak (AM), as well as retention time (RT), were obtained, showing changes in retention times for individual compounds in different chromatograms. Comparing the major statistical parameters generated from datasets of the SpectConnect platform, there are no significant differences. On the other hand, automatic integration in the MestReNova software provides average sensitivity compared to the previously mentioned platforms, while manual correction was able to detect even the smallest peak. When the SIMCA model statistical parameters were compared, there were no big differences between the obtained models when the PCA model for MNOVA-automatic was excluded.

For the Ouachita cultivar sample, an example of the expanded region of the SPME GC-MS chromatogram displays the output data for every tested software solution. Automatic integration without manual correction and TIC checks resulted in the absence of component C and its contribution to the peak of component B. In relation to that, the XCMS platform recognized all three components and expressed their abundance through the base ion peak of each. It can be seen from Figure 1 that, although there is a slight weighting of each of the observed components, the XCMS platform does not recognize this and very directly sets limits for each of them. At the end, the SpectConect platform, using AMDIS deconvolution, separates the superimposed ion currents of the different compounds and gives the total areas for each of them.

Although the parameters of the model and the number of variables as input elements that are analyzed are important when deciding whether to opt for one, it is also necessary to consider the variables that the model recognizes as significant for the separation of certain data sets. Thus, in the mentioned models, a value of 1.3 or more was taken as an elimination parameter for VIP-predictive, and those variables are given in Table 2. A criterion for choosing the limit value for VIP-predictive was a number of variables that were higher than that value in each of the models. A compromise was made between too few and too many variables that were important for separation. Just by simple counting, it is clear that the most significant variables for separation are present in the model with the most input data. The three models based on the SpectConnect platform data, on the other hand, are different in both the number and types of aroma components that separate the two varieties (see Table 2). Nevertheless, the observed differences are not so big. In the OPLS-DA models of the Loch Ness/Columbia star, only E-2-hexenal appeared in the RA dataset as relevant for separation. Except hexanal, hexenal, 2-heptanone, octanal, nonanal, theaspirane B, and ethyl dodecanoate, in the analysis of the Loch Ness/Von OPLS-DA model in VIP-pred, 1-hexanol and 2-heptanol were unique in RA, and 2-heptanol, hexyl acetate, decanal, and 2,6,10-trimethylpentadecane were unique IS datasets. The ethyl tiglate, myrcene, and heptanone were non-characteristic in all the Loch Ness/Nachez models; ethyl-benzaote, α-muurolene, (E)-2-hexenal, (Z)-2-hexen-1-ol, 1,3,8-p-mentatriene, girjunen-β, and (Z)-β-lonone were non-characteristic in Loch Ness/Prime-Ark 45; and decanal, (Z)-calamene, and ethyl dodecanoate were non-characteristic in the Loch Ness/Ouachita OPLS-DA models. As we mentioned, the number of detected and processed variables has decreased, as has the number of potential biomarkers, or, in this case, chemical constituents of aroma that can be defined as characteristics of certain varieties. Even so, this had an effect on the total number of variables with a VIP-pred. score greater than 1.3; however, less than half of them were also called on other platforms. 

Aligning the peaks in MestReNova only by retention times without the possibility of checking the coincidence of their mass spectra leads to the absence of some of the components recognized as deserving of stretching by online platforms while detecting even the smallest peaks increases the probability of finding additional distinguishing markers. All this resulted in the appearance of new variables that were not recognized on any other platform/model.

Regardless of all the challenges presented, common factors can be found in the presented results, which can be unequivocally reliable data that have weight for conclusions. The only question is for what purpose the research is being carried out in order to choose the appropriate approach when designing while keeping in mind all the mentioned facts.

## 4. Conclusions

Regardless of how data were extracted from HS-SPME/GC-MS chromatograms, one must be aware of the limitations of each of the software solutions and present the results accordingly. Although MestReNova offers a relatively simple solution, a lot of manual work is needed to correct irregularities, primarily as a result of overlapping peaks. The XCMS online platform, originally designed for LC-MS data analysis, can also effectively analyze GC-MS data. It should be noted that, for more intense peaks, a lot of *m*/*z* values in the table are obtained, which need to be reduced according to the mass spectrum of the investigated compound. However, due to the algorithm’s nature, fewer represented components are excluded from the matrix and labeled as noise. The SpectConect platform has difficulties processing a large amount of data due to the limitations of the hardware on which it is installed, but, with the application of NIST software, it represents an excellent solution for the metabolomic analyses and identification of individual components in such studies.

## Figures and Tables

**Table 1:** The main statistical parameters of the obtained model with different platforms.

Platform (No. Variables)	Model	No. of Components (pred. + orth. in Y)	R2 X	R2 Y	Q2	CV-ANOVA		Comparison of
						F	p	
XCMS (72)	PCA	6	0.931		0.863			all
	PLS-DA	9	0.950	0.952	0.893	15.05	1 × 10^−31^	all
	OPLS-DA	1 + 2	0.923	0.994	0.963	52.6	6.32 × 10^−9^	Loch Ness/Columbia star
		1 + 1	0.901	0.986	0.978	155.3	1.98 × 10^−11^	Loch Ness/Von
		1 + 3	0.873	0.994	0.963	40.4	5.50 × 10^−7^	Loch Ness/Prime-Ark 45
		1 + 3	0.958	0.993	0.967	45.7	2.89 × 10^−7^	Loch Ness/Nachez
		1 + 1	0.901	0.963	0.944	54.5	5.34 × 10^−8^	Loch Ness/Ouachita
SpectConnect—relative abundance (119)	PCA	6	0.712		0.531			all
	PLS-DA	7	0.729	0.935	0.879	18.2	1 × 10^−31^	all
	OPLS-DA	1 + 1	0.681	0.994	0.979	175.9	2.08 × 10^−12^	Loch Ness/Columbia star
		1 + 1	0.656	0.992	0.981	190.1	1.18 × 10^−12^	Loch Ness/Von
		1 + 2	0.588	0.994	0.961	52.8	2.22 × 10^−8^	Loch Ness/Prime-Ark 45
		1 + 1	0.702	0.991	0.964	101.5	1.14 × 10^−10^	Loch Ness/Nachez
		1 + 1	0.593	0.992	0.971	123.7	2.71 × 10^−11^	Loch Ness/Ouachita
SpectConnect -integrated signal (119)	PCA	6	0.711		0.532			all
	PLS-DA	9	0.761	0.961	0.898	13.9	1 × 10^−31^	all
	OPLS-DA	1 + 1	0.678	0.994	0.981	189.3	1.22 × 10^−12^	Loch Ness/Columbia star
		1 + 1	0.655	0.992	0.981	189.8	1.19 × 10^−12^	Loch Ness/Von
		1 + 2	0.585	0.994	0.962	54.2	1.87 × 10^−8^	Loch Ness/Prime-Ark 45
		1 + 1	0.705	0.99	0.965	103.5	9.81 × 10^−11^	Loch Ness/Nachez
		1 + 1	0.592	0.993	0.973	137	1.29 × 10^−11^	Loch Ness/Ouachita
SpectConnect—base peak (119)	PCA	6	0.706		0.531			all
	PLS-DA	9	0.758	0.958	0.889	13.1	1 × 10^−31^	all
	OPLS-DA	1 + 1	0.67	0.996	0.98	187.9	1.28 × 10^−12^	Loch Ness/Columbia star
		1 + 1	0.64	0.994	0.98	184.3	1.48 × 10^−12^	Loch Ness/Von
		1 + 2	0.582	0.995	0.956	46.8	4.64 × 10^−8^	Loch Ness/Prime-Ark 45
		1 + 1	0.689	0.99	0.964	100.7	1.2 × 10^−10^	Loch Ness/Nachez
		1 + 1	0.61	0.992	0.975	144.7	8.68 × 10^−12^	Loch Ness/Ouachita
MNOVA-automatic (87)	PCA	7	0.610		0.276			all
	PLS-DA	5 + 3	0.687	0.912	0.813	11.0	1 × 10^−31^	all
	OPLS-DA	1 + 2	0.689	0.996	0.952	43.1	7.72 × 10^−9^	Loch Ness/Columbia star
		1 + 1	0.566	0.974	0.923	45.1	3.44 × 10^−8^	Loch Ness/Von
		1 + 1	0.516	0.972	0.934	53.0	1.14 × 10^−8^	Loch Ness/Prime-Ark 45
		1 + 1	0.651	0.983	0.948	68.4	1.90 × 10^−9^	Loch Ness/Nachez
		1 + 2	0.582	0.996	0.964	57.8	1.27 × 10^−8^	Loch Ness/Ouachita
MNOVA-manual corrected (167)	PCA	7	0.698		0.547			all
	PLS-DA	7	0.691	0.923	0.863	18.6	1 × 10^−31^	all
	OPLS-DA	1 + 2	0.766	0.999	0.987	196.3	1.43 × 10^−11^	Loch Ness/Columbia star
		1 + 1	0.667	0.992	0.963	97.4	1.53 × 10^−10^	Loch Ness/Von
		1 + 2	0.542	0.998	0.948	39.9	1.24 × 10^−11^	Loch Ness/Prime-Ark 45
		1 + 1	0.692	0.993	0.961	91.5	2.39 × 10^−10^	Loch Ness/Nachez
		1 + 1	0.605	0.994	0.962	94.8	1.85 × 10^−10^	Loch Ness/Ouachita

**Table 2:** The selected variables (compounds) with VIP-predictive value greater than 1.3. obtained on different platforms.

Platforms	Loch Ness/Columbia Star	Loch Ness/Von	Loch Ness/Prime-Ark 45	Loch Ness/Nachez	Loch Ness/Ouachita
XCMS	Camphene	Ethyl butanoate	Ethyl butanoate	Camphene	Ethyl butanoate
	Benzaldehyde	2-Heptanone	(E)-2-Hexenal	Limonene	(E)-2-Hexenal
	Limonene	Limonene	1-Hexanol	Nonanal	2-Heptanone
	Acetophenone	Nonanal	2-Heptanone	α-Cubebene	β-pinene
	Terpinolene	Theaspirane A	Camphene	Sibirene	Limonene
	Linalol	Theaspirane B	(E)-2-Heptenal	(Z)-Calamenene	Nonanal
	Nonanal	(Z)-Calamenene	(E)-(3,3-Dimethyl-cyclohexyl-idene)acetaldehyde	α-Calacorene	(Z)-Carveol
	Camphor	Ethyl dodecanoate	Acetophenone		Theaspirane A
	1-Nonanol		Camphor		Theaspirane B
	Citronellol		1-Nonanol		α-Cubebene
	Carvone		p-Cymen-8-ol		Sibirene
	Carvacrol		Carvone		(Z)-Calamenene
			Theaspirane A		Ethyl dodecanoate
			Theaspirane B		
			2,2,4,4,6,8,8-Heptamethylnonane		
			(Z)-β-Ionone		
SpectConnect—relative abundance	(E)-2-Hexenal	(E)-2-Hexenal	Hexenal	2-Heptanone	Pentanal
	1-Heptanol	1-Hexanol	1-Hexanol	2-Heptanol	Hexanal
	Octanal	2-Heptanone	1-Heptanol	Ethyl tiglate	(E)-2-Hexenal
	δ-Carene	2-Heptanol	Camphor	Myrcene	1-Hexanol
	Limonene	Octanal	Methyl benzoate	Nonanal	2-Heptanone
	Acetophenone	Nonanal	Ethylbenzoate	(Z)-Carveol	(E)-2-Heptenal
	Linalol	Theaspirane B	(Z)-Carveol	α-Copaene	1-Heptanol
	Nonanal	Ethyl dodecanoate	Carvone	Ethyl decanoate	Octanal
	Camphor		Theaspirane B	(Z)-Calamenene	Nonanal
	Methyl salicylate		β-Gurjunene		(Z)-Carveol
	Carvone		(Z)-β-Ionone		Theaspirane B
	(Z)-β-Ionone		α-Muurolene		(Z)-Calamenene
					Ethyl dodecanoate
SpectConnect—integrated signal	Hexanal	Hexanal	Hexanal	Heptanone	Hexanal
	1-Heptanol	(E)-2-Hexenal	(E)-2-Hexenal	2-Heptanol	(E)-2-Hexenal
	Octanal	2-Heptanone	(Z)-2-Hexen-1-ol	Nonanal	1-Hexanol
	δ-Carene	2-Heptanol	1-Hexanol	(Z)-Carveol	2-Heptanone
	Limonene	Octanal	1-Heptanol	α-Copaene	(E)-2-Heptenal
	Acetophenone	Hexyl acetate	Methyl benzoate	Ethyl dodecanoate	1-Heptanol
	Linalol	Nonanal	1,3,8-p-Menthatriene	(Z)-Calamenene	Octanal
	Nonanal	Decanal	Camphor		Nonanal
	Camphor	Theaspirane B	(Z)-Carveol		Decanal
	Methyl salicylate	2,6,10-Trimethylpentadecane	Carvone		(Z)-Carveol
	Carvone		Theaspirane B		Theaspirane B
	(Z)-β-Ionone		β-Gurjunene		Ethyl dodecanoate
			(Z)-β-Ionone		
SpectConnect—base peak	Hexanal	Hexanal	Hexanal	2-Heptanone	Pentanal
	1-Heptanol	Hexenal	(E)-2-Hexenal	Nonanal	Hexanal
	Octanal	2-Heptanone	(Z)-2-Hexen-1-ol	(Z)-Carveol	(E)-2-Hexenal
	δ-Carene	Octanal	1-Hexanol	α-Copaene	1-Hexanol
	Limonene	Nonanal	1-Heptanol	Ethyl dodecanoate	2-Heptanone
	Acetophenone	Theaspirane B	Methyl benzoate	(Z)-Calamenene	(E)-2-Heptenal
	Linalol	Ethyl dodecanoate	1,3,8-p-Menthatriene		1-Heptanol
	Nonanal		Camphor		Octanal
	Camphor		(Z)-Carveol		Nonanal
	Methyl salicylate		Carvone		(Z)-Carveol
	Carvone		Theaspirane B		Theaspirane B
	(Z)-β-Ionone		β-Gurjunene		(Z)-Calamenene
			(Z)-β-Ionone		Ethyl dodecanoate
MNOVA-automatic	2-Heptanone	(E)-2-Hexenal	Ethyl (E)-2-butenoate	2-Heptanone	Ethyl (E)-2-butenoate
	2-Heptanol	Hexyl acetate	(E)-2-Hexenal	2-Heptanol	(E)-2-Hexenal
	(E)-2-Heptenal	Linalol	Hexyl acetate	Camphene	1-Hexanol
	Octanal	(Z)-3-Nonen-1-ol	(E)-2-Nonenal	Myrcene	(E)-2-Heptenal
	Hexyl acetate	Decanal	Borneol	Hexyl acetate	β-pinene
	2-Ethyl-1-Hexanol	α-Muurolene	1-Nonanol	γ-Terpinene	Hexyl acetate
	γ-Terpinene				
	Ethyl 2-hydroxy-4-methylpentanoate	4-Phenyldodecane	Methyl salicylate	Myrtenol	Linalol
	Linalol		Carvone	α-Copaene	Myrtenol
	(E)-2-Nonenal		Theaspirane A	(Z)-Calamenene	Decanal
	1-Nonanol		Theaspirane B		
	Decanal				
	Carvone				
	(E)-Caryophyllene				
	(Z)-β-Ionone				
	Heptadecane				
MNOVA manual corrected	2-Heptanone	(E)-2-Hexenal	(E)-2-Hexenal	2-Heptanone	Ethyl butanoate
	Camphene	2-Heptanone	Ethyl 4-methylpentanoate	Ethyl tiglate	(E)-2-Hexenal
	Ethyl 2-hydroxy-4-methylpentanoate	Nonanal	1-Heptanol	Camphene	2-Heptanone
	Linalol	Decanal	Hexyl acetate	Myrcene	2-Heptanol
	Nonanal	Nonanoic acid	3-Ethyl-4-methyl-1-pentanol	Hexyl acetate	(E)-2-Heptenal
	1-Nonanol	Theaspirane A	Methyl benzoate	Nonanal	β-pinene
	α-Terpineol	Theaspirane B	Camphor	Methyl chavicol	Hexanoic acid
	Decanal	α-Copaene	Borneol	Verbenone	Hexyl acetate
	Verbenone	2,6,10-Trimethyltridecane	1-Nonanol	α-Cubebene	Decanal
	Citronellol	Alloaromadendrene	Citronellol	γ-Nonalactone	Citronellol
	Geraniol	γ-Himachalene	Carvone	α-Ylangene	Theaspirane A
	Dehydro-ar-ionene	Ethyl dodecanoate	Theaspirane A	α-Copaene	Theaspirane B
	(E)-Caryophyllene		Theaspirane B	Ethyl decanoate	γ-Nonalactone
	α-Ionone		α-Ylangene	Sibirene	α-Copaene
	1-Dodecanol		α-Copaene	γ-Himachalene	β-Gurjunene
	(Z)-β-Ionone		(E)-Caryophyllene	(Z)-Calamenene	2,6,10-Trimethyltridecane
			β-Gurjunene		(Z)-Calamenene
			2,6,10-Trimethyltridecane		Ethyl dodecanoate
			(Z)-β-Ionone		
			10,11-epoxy-Calamenene		

## Data Availability

The original contributions presented in the study are included in the article/supplementary material, further inquiries can be directed to the corresponding author.

## Supplementary Materials

The following supporting information can be downloaded at: https://www.mdpi.com/article/10.3390/foods13081222/s1, Table S1: The HS-SPME/GC-MS aroma analyses of six blackberry varieties.

## References

[1] Everitt B.S. (1975). Multivariate Analysis: The Need for Data, and Other Problems. Br. J. Psychiatry.

[2] Goldrick S., Sandner V., Cheeks M., Turner R., Farid S.S., McCreath G., Glassey J. (2020). Multivariate Data Analysis Methodology to Solve Data Challenges Related to Scale-Up Model Validation and Missing Data on a Micro-Bioreactor System. Biotechnol. J..

[3] Dempster A.P. (1971). An overview of multivariate data analysis. J. Multivar. Anal..

[4] Nicholson J.K., Connelly J., Lindon J.C., Holmes E. (2002). Metabonomics: A platform for studying drug toxicity and gene function. Nat. Rev. Drug Discov..

[5] Verpoorte R., Choi Y.H., Mustafa N.R., Kim H.K. (2008). Metabolomics: Back to basics. Phytochem. Rev..

[6] Wolfender J.-L., Rudaz S., Hae Choi Y., Kyong Kim H. (2013). Plant Metabolomics: From Holistic Data to Relevant Biomarkers. Curr. Med. Chem..

[7] Shuman J.L., Cortes D.F., Armenta J.M., Pokrzywa R.M., Mendes P., Shulaev V. (2011). Plant Metabolomics by GC-MS and Differential Analysis. Plant Reverse Genet. Methods Protoc..

[8] Worley B., Powers R. (2012). Multivariate Analysis in Metabolomics. Curr. Metabolomics.

[9] Stoudt S., Vásquez V.N., Martinez C.C. (2021). Principles for data analysis workflows. PLoS Comput. Biol..

[10] Niu W., Knight E., Xia Q., McGarvey B.D. (2014). Comparative evaluation of eight software programs for alignment of gas chromatography–mass spectrometry chromatograms in metabolomics experiments. J. Chromatogr. A.

[11] Maciel E.V.S., Pereira dos Santos N.G., Vargas Medina D.A., Lanças F.M. (2022). Electron ionization mass spectrometry: Quo vadis?. Electrophoresis.

[12] Medeiros P.M. (2018). Gas Chromatography–Mass Spectrometry (GC–MS). Encyclopedia of Geochemistry.

[13] Fiehn O. (2016). Metabolomics by Gas Chromatography–Mass Spectrometry: Combined Targeted and Untargeted Profiling. Curr. Protoc. Mol. Biol..

[14] Coble J.B., Fraga C.G. (2014). Comparative evaluation of preprocessing freeware on chromatography/mass spectrometry data for signature discovery. J. Chromatogr. A.

[15] Styczynski M.P., Moxley J.F., Tong L.V., Walther J.L., Jensen K.L., Stephanopoulos G.N. (2007). Systematic Identification of Conserved Metabolites in GC/MS Data for Metabolomics and Biomarker Discovery. Anal. Chem..

[16] Hamany Djande C.Y., Pretorius C., Tugizimana F., Piater L.A., Dubery I.A. (2020). Metabolomics: A Tool for Cultivar Phenotyping and Investigation of Grain Crops. Agronomy.

[17] O’Hagan S., Dunn W.B., Brown M., Knowles J.D., Kell D.B. (2005). Closed-Loop, Multiobjective Optimization of Analytical Instrumentation: Gas Chromatography/Time-of-Flight Mass Spectrometry of the Metabolomes of Human Serum and of Yeast Fermentations. Anal. Chem..

[18] Fialkov A.B., Steiner U., Lehotay S.J., Amirav A. (2007). Sensitivity and noise in GC–MS: Achieving low limits of detection for difficult analytes. Int. J. Mass Spectrom..

[19] Stein S.E. (1999). An integrated method for spectrum extraction and compound identification from gas chromatography/mass spectrometry data. J. Am. Soc. Mass Spectrom..

[20] Ibáñez E., López-Sebastián S., Ramos E., Tabera J., Reglero G. (1998). Analysis of volatile fruit components by headspace solid-phase microextraction. Food Chem..

[21] D’Agostino M.F., Sicari V., Giuffrè A.M., Soria A.C. (2022). Blackberries (Rubus ulmifolius Schott) from Calabria (Italy): A comprehensive characterisation. Eur. Food Res. Technol..

[22] Avramidou E., Sarri E., Ganopoulos I., Madesis P., Kougiteas L., Papadopoulou E.-A., Aliferis K.A., Abraham E.M., Tani E. (2023). Genetic and Metabolite Variability among Commercial Varieties and Advanced Lines of *Vicia faba* L.. Plants.

[23] Padilla-Jimenez S.M., Angoa-Pérez M.V., Mena-Violante H.G., Oyoque-Salcedo G., Renteria-Ortega M., Oregel-Zamudio E. (2019). Changes in the Aroma of Organic Blackberries (*Rubus Fruticosus*) During Ripeness. Anal. Chem. Lett..

[24] Dayananda B., Owen S., Kolobaric A., Chapman J., Cozzolino D. (2023). Pre-processing Applied to Instrumental Data in Analytical Chemistry: A Brief Review of the Methods and Examples. Crit. Rev. Anal. Chem..

[25] Oliveri P., Malegori C., Simonetti R., Casale M. (2019). The impact of signal pre-processing on the final interpretation of analytical outcomes—A tutorial. Anal. Chim. Acta.

[26] Duan Y., Yang H., Wei Z., Yang H., Fan S., Wu W., Lyu L., Li W. (2023). Effects of Different Nitrogen Forms on Blackberry Fruit Quality. Foods.

[27] Wu Y., Huang X., Yang H., Zhang S., Lyu L., Li W., Wu W. (2023). Analysis of flavonoid-related metabolites in different tissues and fruit developmental stages of blackberry based on metabolome analysis. Food Res. Int..

[28] Zhang M.-Q., Zhang J., Zhang Y.-T., Sun J.-Y., Prieto M.A., Simal-Gandara J., Putnik P., Li N.-Y., Liu C. (2023). The link between the phenolic composition and the antioxidant activity in different small berries: A metabolomic approach. LWT.

[29] Kodikara C., Netticadan T., Bandara N., Wijekoon C., Sura S. (2024). A new UHPLC-HRMS metabolomics approach for the rapid and comprehensive analysis of phenolic compounds in blueberry, raspberry, blackberry, cranberry and cherry fruits. Food Chem..

[30] Kim M.J., Lee M.Y., Shon J.C., Kwon Y.S., Liu K.-H., Lee C.H., Ku K.-M. (2019). Untargeted and targeted metabolomics analyses of blackberries–Understanding postharvest red drupelet disorder. Food Chem..

[31] Columbia Star Thornless Blackberry. https://raintreenursery.com/products/columbia-star-thornless-blackberry-4-inch-pot.

[32] Loch Ness Thornless Blackberry. https://raintreenursery.com/products/loch-ness-thornless-blackberry-2yr-bareroot.

[33] Natchez Thornless Blackberry Plant. https://www.starkbros.com/products/berry-plants/blackberry-plants/natchez-thornless-blackberry.

[34] Ouachita Thornless Blackberry Plant. https://www.starkbros.com/products/berry-plants/blackberry-plants/ouachita-thornless-blackberry.

[35] Prime-Ark® 45 Primocane Blackberry Plant. https://www.starkbros.com/products/berry-plants/blackberry-plants/prime-ark-45-primocane-blackberry.

[36] Von Blackberry Plant. https://www.isons.com/shop/berry-plants/blackberry/von-blackberry-plant/.

[37] XCMS TM. https://xcmsonline.scripps.edu/landing_page.php?pgcontent=mainPage.

[38] SpectConnect. http://spectconnect.mit.edu/index.php.

[39] Dervishi E., Zhang G., Zwierzchowski G., Mandal R., Wishart D.S., Ametaj B.N. (2020). Serum metabolic fingerprinting of pre-lameness dairy cows by GC–MS reveals typical profiles that can identify susceptible cows. J. Proteomics.

[40] Smith C.A., Want E.J., O’Maille G., Abagyan R., Siuzdak G. (2006). XCMS: Processing Mass Spectrometry Data for Metabolite Profiling Using Nonlinear Peak Alignment, Matching, and Identification. Anal. Chem..

[41] Ivanović S., Simić K., Tešević V., Vujisić L., Ljekočević M., Gođevac D. (2021). GC-FID-MS Based Metabolomics to Access Plum Brandy Quality. Molecules.

